# Intestinal Microbiota - An Unmissable Bridge to Severe Acute Pancreatitis-Associated Acute Lung Injury

**DOI:** 10.3389/fimmu.2022.913178

**Published:** 2022-06-14

**Authors:** Zhengjian Wang, Fan Li, Jin Liu, Yalan Luo, Haoya Guo, Qi Yang, Caiming Xu, Shurong Ma, Hailong Chen

**Affiliations:** ^1^ Department of General Surgery, The First Affiliated Hospital of Dalian Medical University, Dalian, China; ^2^ Institute (College) of Integrative Medicine, Dalian Medical University, Dalian, China; ^3^ Laboratory of Integrative Medicine, The First Affiliated Hospital of Dalian Medical University, Dalian, China; ^4^ Department of Traditional Chinese Medicine, The Second Affiliated Hospital of Dalian Medical University, Dalian, China; ^5^ Department of Molecular Diagnostics and Experimental Therapeutics, Beckman Research Institute of City of Hope Comprehensive Cancer Center, Duarte, CA, United States

**Keywords:** severe acute pancreatitis-associated lung injury, intestinal microbiota, microbiota-gut-lung axis, bacterial translocation, pathogen-associated molecular patterns, immune imbalance

## Abstract

Severe acute pancreatitis (SAP), one of the most serious abdominal emergencies in general surgery, is characterized by acute and rapid onset as well as high mortality, which often leads to multiple organ failure (MOF). Acute lung injury (ALI), the earliest accompanied organ dysfunction, is the most common cause of death in patients following the SAP onset. The exact pathogenesis of ALI during SAP, however, remains unclear. In recent years, advances in the microbiota-gut-lung axis have led to a better understanding of SAP-associated lung injury (PALI). In addition, the bidirectional communications between intestinal microbes and the lung are becoming more apparent. This paper aims to review the mechanisms of an imbalanced intestinal microbiota contributing to the development of PALI, which is mediated by the disruption of physical, chemical, and immune barriers in the intestine, promotes bacterial translocation, and results in the activation of abnormal immune responses in severe pancreatitis. The pathogen-associated molecular patterns (PAMPs) mediated immunol mechanisms in the occurrence of PALI *via* binding with pattern recognition receptors (PRRs) through the microbiota-gut-lung axis are focused in this study. Moreover, the potential therapeutic strategies for alleviating PALI by regulating the composition or the function of the intestinal microbiota are discussed in this review. The aim of this study is to provide new ideas and therapeutic tools for PALI patients.

## Introduction

Severe acute pancreatitis (SAP) is a serious abdominal disease with multiple complications and high mortality rates with an average annual incidence of about 6.75 cases per 100,000 people worldwide. Systemic inflammatory response syndrome (SIRS), multiple organ dysfunction syndromes (MODS), and sepsis are frequently associated with it (1, 2). Acute lung injury (ALI), the most common and earliest organ dysfunction during SAP, is the leading cause of death in SAP patients, particularly in elderly SAP patients (60%-70%) (3–5). Multi-mechanisms mediated crosstalk among the pancreatic necrosis, bacteremia, intestinal barrier failure, etc. are the reported reasons for the pathological process of SAP-associated lung injury (PALI) (6). However, the pathogenesis of PALI has not been fully elucidated to date, partly attributed to the lack of effective treatment strategies as well as a longer treatment cycle, unsatisfying prognosis, and economic burden of the disease (7, 8).

In recent years, with the in-depth research in terms of the gut microbiota, their critical roles in holding the intestinal immune barrier, and the defense functions are increasingly revealed. In PALI, the molecular mechanisms by which the gut microbiota mediate PALI have become a hot topic (9–11). Trillions of microorganisms (including bacteria, fungi, and viruses) are present in the host intestine, and they perform a variety of regulatory effects on the physiological activities through their metabolites, or the signaling pathways (axis), symbiotically co-existing with the host (12). A microecological balance is achieved between host and microorganisms under a healthy state, which is referred to as homeostasis, and normal physiological functions are maintained in the host. However, external factors (such as infection, toxins, dietary changes, disease, etc.) might alter or disrupt the balance between them and the disturbance of intestinal microbiota will in turn induce or accelerate disease progression (13).

Studies on the microbiota-gut-lung axis have enhanced our understanding of PALI, and current research has pointed out that bidirectional communication exists between the lung and gut microbiota, with intestinal microorganisms and their metabolites playing key roles (14). Moreover, opportunistic pathogenic microbes in the intestine and their PAMPs are metronomes in the pathogenesis of ALI (15). Intestinal microbes are prospective targets in the treatment and control of PALI.

**Figure 1:**
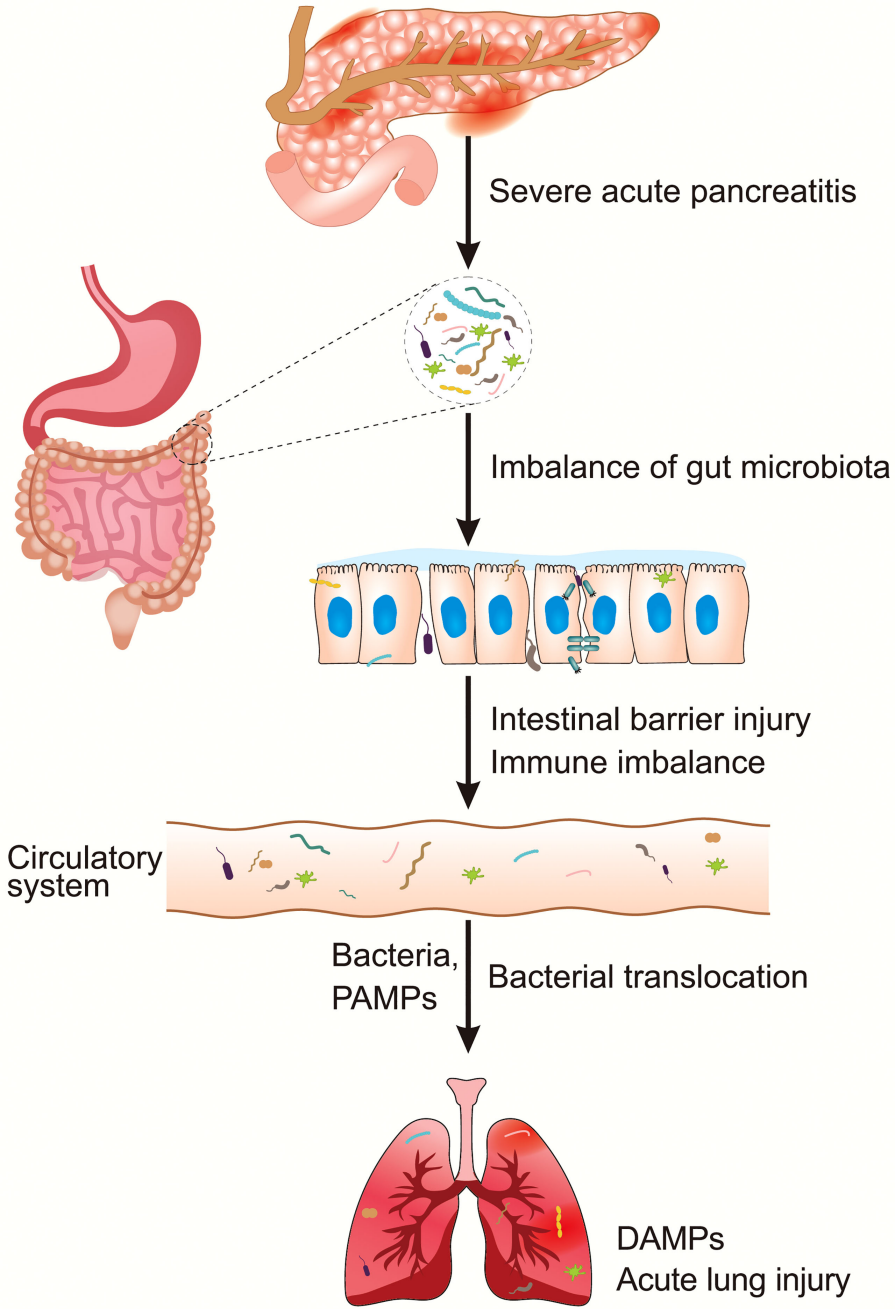
An overview of intestinal microbiota dysbiosis in PALI. DAMPs, damage-associated molecular patterns; PALI, severe acute pancreatitis-associated acute lung injury; PAMPs, pathogen-associated molecular patterns.

However, to the best of our knowledge, reviews on the involvement of intestinal microbiota in the pathogenies of PALI are lacking. In this paper, we first describe the structural and species alterations of the intestinal microbiota during SAP, which causes intestinal barrier injury, including physical, chemical, and immune barrier disruptions. Then, the possible pathways of barrier injury-induced bacterial translocation under SAP, and the mechanisms of bacterial translocation-associated ALI, are discussed. Specifically, the pathogen-associated molecular patterns (PAMPs) mediated immunol mechanisms in the occurrence of PALI by binding with pattern recognition receptors (PRRs) *via* the microbiota-gut-lung axis are focused. The diagram of summary contents of this paper is illustrated in **Figure 1**. Finally, this paper summarizes the prospective intervention strategies for the treatment of PALI mediated by the regulation of intestinal microbiota as well, which is expected to provide references in clinical practice in the future.

## Dysbiosis of Intestinal Microbiota in SAP

With the development of high-throughput sequencing techniques, several studies have proven that the abundance, as well as the diversity, of the intestinal microbiota are significantly altered during SAP (**Table 1**). The diversity of intestinal microbiota is reduced in SAP patients in comparison to the healthy controls, indicating a tendency of obligate anaerobic bacteria to be replaced by facultative anaerobic bacteria as the dominant bacteria in the intestine. At the phylum level, the abundance of Proteobacteria and Actinobacteria is elevated in the intestine of SAP patients, whereas the abundance of Firmicutes and Bacteroides is decreased. At the genus level, SAP patients show a significant increase in the abundance of facultative anaerobic bacteria in the intestine, such as *Escherichia*, *Enterococcus*, *Enterobacter*, *Streptococcus*, etc., with a decrease in *Bacteroides*, *Bifidobacterium*, and *Blautia*, etc., and the SAP animal models show similar results (2, 16–18, 20, 21). Furthermore, analyses of the bacterial community reveal that the most noticeable differences in species abundance are located in the cecum and colon (24).

**Table 1:** Intestinal microbiota dysbiosis in SAP.

Species	Sample	Sequencing methods	Increased bacteria in abundance	Decreased bacteria in abundance	References
Human	Feces	16S rRNA sequencing	*Escherichia-Shigella, Enterococcus*	*Dorea longicatena*, *Blautia wexlerae*, *Bacteroides Ovatus*	Hu et al. (16)
Human	Feces	Metagenomics sequencing	*Enterococcus*	*Blautia*	Yu et al. (17)
Human	Rectal swab	16S rRNA sequencing	*Enterococcus*	*Eubacterium halli*, *Blautia*	Yu et al. (18)
Human	Blood	16S rDNA sequencing	*Bacteroides*, *Stenotrophomonas*, *Serratia*, *Rhizobium*, *Prevotella*, *Staphylococcus*, *Paracoccus *	*Acinetobacter*, *Lactococcus*, *Flavobacterium, Pseudomonas, Corynebacterium, Sphingobium*	Li et al. (19)
Mice	Feces	16S rRNA sequencing	*Prevotellaceae, Epsilonproteobacteria, Proteobacteria*	*Firmicutes*, *Clostridiales*	Jin et al. (2)
Mice	Feces	16S rRNA sequencing	*Proteobacteria*, *Escherichia/Shigella*, *Streptococcus *	*N/A*	Van et al. (20)
Mice	Feces	16S rRNA sequencing	*Escherichia-Shigella*, *Enterococcus*	*Akkermansia*	Mei et al. (21)
Mice	Feces	16S rRNA sequencing	*Escherichia–Shigella*, *Acinetobacter*, *Stenotrophomonas*, *Geobacillus*	*Blautia*, *Bacteroides*, *Alloprevotella*, *Gemella*	Zhu et al. (22)
Rats	Feces	16S rRNA sequencing	*Helicobacter*, *Escherichia-Shigella*	*Lactobacillus*, *Prevotella*	Piao et al. (11)
Rats	Feces	qPCR	*Escherichia*	*Lactobacillus*, *Bifidobacterium*	Su et al. (23)

## Dysbiosis of Gut Microbiota Damages the Intestinal Barrier

**Figure 2:**
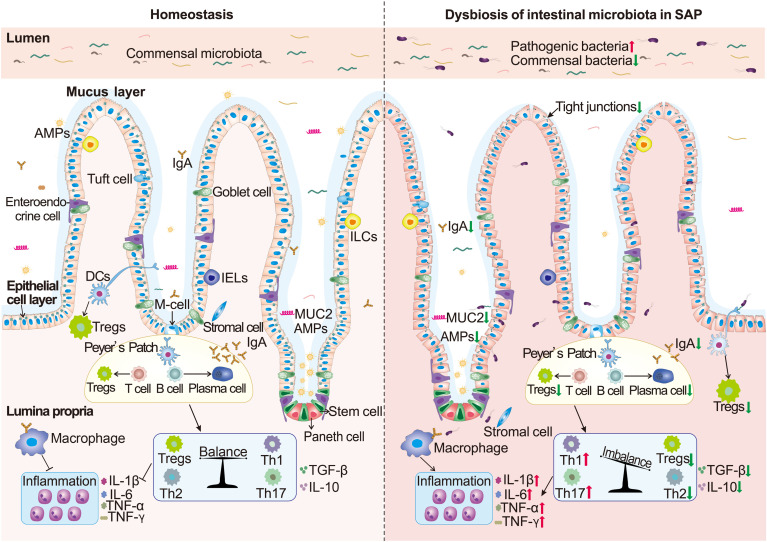
The mechanisms of intestinal barrier disruption mediated by intestinal bacterial dysbiosis. The increase of intestinal pathogenic bacteria and the decrease of commensal metabolites in severe acute pancreatitis (SAP) jointly lead to the thinning of the intestinal mucus layer and the decrease of antimicrobial peptides (AMPs), mucin2 (MUC2), and IgA (SIgA) secretion, resulting in direct contact of pathogenic bacteria with the intestinal epithelial cells (IECs) and an “inflammatory storm”. The abnormal immune responses further lead to IEC damage, slow renewal, and disruption of the tight junctions (TJs) in epithelial cells, further stimulating excessive pro-inflammatory factors on the intestinal mucosal immune system. Finally, the integrity of the intestinal barrier is destroyed, bacteria and products translocate to the lungs, and then PALI ensues.

The 100 trillion microorganisms in the gut are separated from the host by physical, chemical, and immune barriers of the intestine (25). Current studies have pointed out that the damage to the intestinal barrier integrity caused by the dysfunction of intestinal microecology during SAP primarily contributes to the development of PALI (26–30). The diagram depicting the mechanism is summarized in **Figure 2**.

### Intestinal Chemical Barrier Integrity

The chemical barrier in the intestine is mainly composed of the mucus layer on the surface of the intestinal epithelium, which contains digestive fluids (gastric acid, intestinal fluid, bile, etc.) and mucus secreted from goblet cells (GCs), and the antimicrobial peptides (AMPs) that are secreted from paneth cells (PCs) in intestinal epithelial cells (IECs) (31, 32). The intestinal mucus layer develops the first line defense of the intestine against pathogens (33–35). The secretion of mucus is regulated by microbes as well. As a case in point, the obligate anaerobic bacteria could induce the secretion of AMPs and raise the density of GCs and the production of mucin 2 through their metabolites (acetic acid) in order to strengthen the solidity of the barrier (36).

In the early stages of SAP, the mucus layer begins to disrupt, and it is observed that the mRNA levels of lysozyme and α-defensin are reduced (37–39). Increased colonization of opportunistic pathogenic bacteria in the ileum, such as *Escherichia coli*, *Streptococcus*, *Proteus*, and *Enterococcus*, could lead to a reduction in AMPs and the possibility of pathogenic bacteria directly penetrating the mucus layer. Then, the bacterial translocation (BT) in the intestine (especially the ileum) will lead to injury of the intestinal epithelial cells (IECs) (17). With the progress of SAP, large amounts of Ca^2+^ and fatty acids (butyric acid) are consumed, resulting in a higher pH environment in the intestine, which inhibits the survival of probiotics, leading to a further reduction in the repairment of chemical barrier integrity (31, 40, 41).

### Intestinal Physical Barrier Integrity

The physical barrier of the intestine is made up of a layer of IECs that includes the columnar epithelium and cell gap junction (31). The major IECs, namely enterocytes, GCs, PCs, and enteroendocrine cells, are differentiated from the progenitor cells derived from the Lgr5^+^ intestinal stem cells located in the Lieberkühn crypt, and IECs could be updated every 3-5 days (42). The physical barrier separates the bacteria from the lamina propria immune cells and performs sensory/defense functions or immune tolerance through PRRs mediated by PAMPs on the microbial surface (43, 44). Cell junctions include tight junctions (TJs), adhesion junctions, and desmosomes, with TJs being the main mode, including claudin, occludin, tricellulin, zonula occludens-1 (ZO-1), etc., and they form an osmotic barrier, close the gap between IECs, and maintain the polarity of cells (45).

The disruption of the intestinal physical barrier during SAP is primarily attributed to an imbalance in the anaerobic bacteria with pathogenic bacteria as the dominant bacteria. *Lactobacillus reuteri*, the dominant anaerobic bacterium in the gut, can stimulate the proliferation and differentiation of Lgr5^+^ cells, reducing the levels of proinflammatory cytokines, serum lipopolysaccharide (LPS), and peptidoglycan, and restoring damaged intestinal epithelia. In addition, the abundance of *Bifidobacterium* in the intestine is positively correlated with the expression of occludin, claudin-1, and ZO-1 in TJs (46, 47). When the dysbiosis of microorganisms occurs, reduction in the dominant anaerobic bacteria causes TJs injury and increased permeability in the intestine. Meanwhile, the increased pathogenic bacteria will activate PAMPs, leading to a persistent and overwhelming “inflammatory storm” on the intestinal cell surface through the activation of mitogen-activated protein kinase (MAPK) or nuclear factor-kappa B (NF-κB) pathways, resulting in the necrosis of epithelial cells and the shedding of TJs. Then, the intestinal pathogens in the lamina propria could activate the damage-associated molecular patterns (DAMPs), which cause abnormal immune responses in the intestine (48–50).

### Intestinal Immune Barrier Integrity

IECs, intestinal intraepithelial lymphocytes (IELs), lamina propria lymphocytes (LPLs), Peyer patch (PP), mesenteric lymph nodes (MLNs), as well as secretory IgA (SIgA) from plasma cells make up the intestinal immune barrier (25, 51). As the last line of defense against pathogenic bacteria, the intestinal immune system maintains a delicate dynamic balance with the commensal bacteria (52).

The intestinal microbiota has been found to be a central hub for the development and regulation of the host’s innate and adaptive immune system (53). Germ-free (GF) mice have severe immunodeficiencies, manifested mainly in the reduction of gut-associated lymphoid tissues (GALTs), Th17 cells, regulatory T cells (Tregs), plasma cells, and epithelial CD8^+^ T cells (54). *Bacterooides fragilis*, *Lactobacilli*, and *Bacteroides thetaiotaomicron*, etc., could directly modulate Tregs, Th1 cells, Th17 cells, plasma cells, and intervene in the intestinal immune homeostasis (55–57). During SAP, an increase in *Salmonella*, *Escherichia coli*, *Enterococcus*, and *Proteus* could promote the secretion of pro-inflammatory cytokines (IL-1, IL-6, TNF-α, IFN-γ, etc.) by dendritic cells (DCs) and macrophages, mediated by PAMPs, resulting in the differentiation of immature T cells into Th1/Th17 cells and the subsequent immune responses (58). Moreover, the proliferation of pathogenic bacteria exerts an inhibitory effect on Tregs, Th2 cells, and B cells, which causes a decrease in the secretion of SIgA and anti-inflammatory cytokines (IL-10/TGF-β), and results in a continuous “pathological” activation of immune responses and a “suicidal” attack in the intestine (58–60).

### Injury of Barrier Functions by Changes in Bacterial Metabolites

Numerous studies have demonstrated that bacterial metabolites are involved in the functions of the intestinal barrier (61). For instance, short-chain fatty acids (SCFAs), generated by *Bifidobacterium*, *Bacteroides*, *Brucella*, and *Clostridium*, can enhance the physical and immune barriers of the gut in a variety of ways, such as inhibiting the proliferation of harmful bacteria, promoting the recovery of IECs, enhancing the protein expression of TJs, and maintaining host immune homeostasis, etc., thereby making them the “loyal guardians” (62, 63). Furthermore, deoxycholic acid (DCA), a secondary bile acid metabolite produced by *Bacteroides*, enhances the gut physical barrier by inhibiting the renewal and repair of IECs by activating Farnesoid X receptor (FXR) (64). Indole-3-ethanol, a metabolite of tryptophan produced by *Lactobacillus*, boosts the function of TJs by binding the arylhydrocarbon receptor (AhR) (65). Trimethylamine N-oxide (TMAO) formed from dietary choline promotes the maturation and secretion of IL-1β and IL-18 mediated by nucleotide-binding oligomerization domain-like receptors (NLRs), consequently regulating intestinal inflammation (66).

## Pathways of Bacterial Translocation to the Lung

It is observed that certain bacteria from the lower gastrointestinal tract are enriched in SAP lungs and this phenomenon is referred to as bacterial translocation (BT) (67–69). BT is described as the process by which bacteria and/or their products pass through the intestinal barrier and enter the systemic circulation, tissues, or organs (such as lungs) by MLNs and the portal system, and is thought to be the primary reason for PALI, systemic sepsis, and MODS in patients with SAP (70). Also, lower levels of barrier proteins including occludin, ZO-1, and activation of the platelet-activating receptor (PAFr) are observed to be accompanied by BT (71, 72).

**Figure 3:**
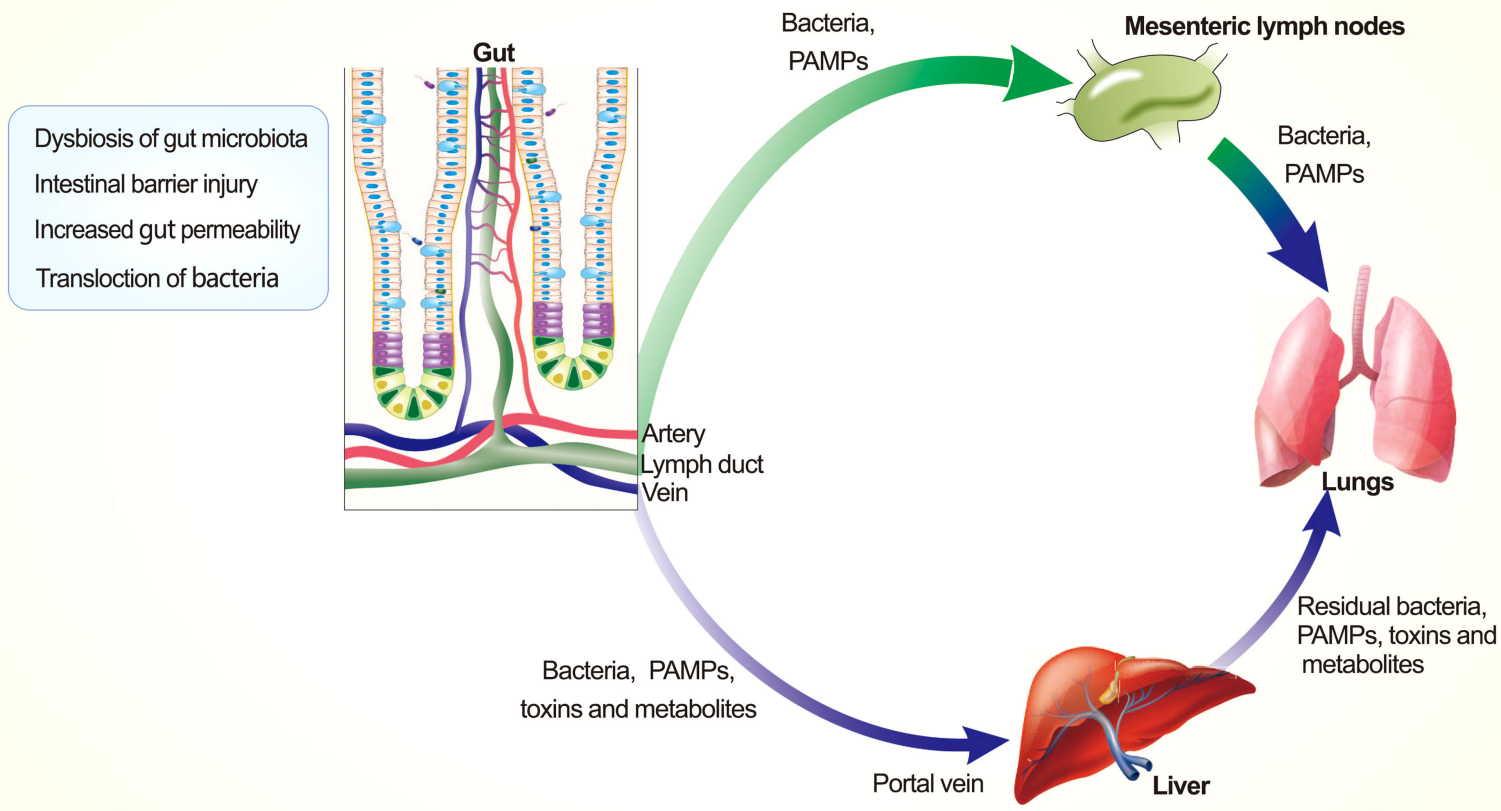
Pathways of intestinal bacterial translocation. Intestinal pathogenic bacteria, PAMPs, and metabolites translocate to the lung *via* lymphatic route and blood circulatory system.

In recent years, high-throughput sequencing techniques have deepened our understanding of BT, the incidence of which is closely linked to the severity of SAP and the degree of bacterial imbalance (19, 73–75). Current studies have suggested that intestinal BT to the lung is primarily mediated by lymphatic and hematogenous dissemination, as summarized in **Figure 3**, and BT is proven to be a facilitator, rather than a trigger for PALI (76). The thoracic duct allows the mesenteric lymphatic fluid to enter the circulatory system, and the lungs are the first exposure points to those pathogens in the pulmonary circulation. Injecting mesenteric lymphatic fluid (from SAP rats) into the veins of healthy rats could trigger PALI, and mesenteric lymphatics ligation or accelerating its excretion can help alleviate inflammation symptoms in rat models (77–80). Moreover, danger-associated molecular patterns (DAMPs) released from lymphatics play a regulatory role in eliciting the ALI (81).

In the early stage of SAP, the kupffer cell forms a “firewall” in the liver that is able to remove the majority of pathogens and PAMPs from the bloodstream. With the increase in pathogenic bacteria, the intestinal inflammatory storm is initiated, leading to the disordered immune system and impaired intestinal barrier integrity, which results in the accelerated shift of microbes and their PAMPs, and then the intestinal toxins enter the vena cava and induce PALI (82, 83). In the late stage of SAP, a substantial amount of ascites is produced, and the pathogenic bacteria and PAMPs will cause bacterial peritonitis following the “fermentation” in the peritoneal cavity and then enter the circulatory system *via* the mesenteric lymphatic system or visceral capillaries to cause ALI (84). However, the choice and timing of abdominal puncture drainage (APD) in SAP patients is controversial. Early APD is recommended as an effective adjuvant in SAP patients by the current American Gastroenterological Association (AGA) guidelines for the management of necrotizing pancreatitis (85). While some scholars claim that APD does not decrease the overall risks of mortality in SAP patients, the mechanisms of APD in PALI still need to be further investigated (86, 87).

## Mechanisms of ALI Caused by Intestinal Bacterial Dysbiosis

**Figure 4:**
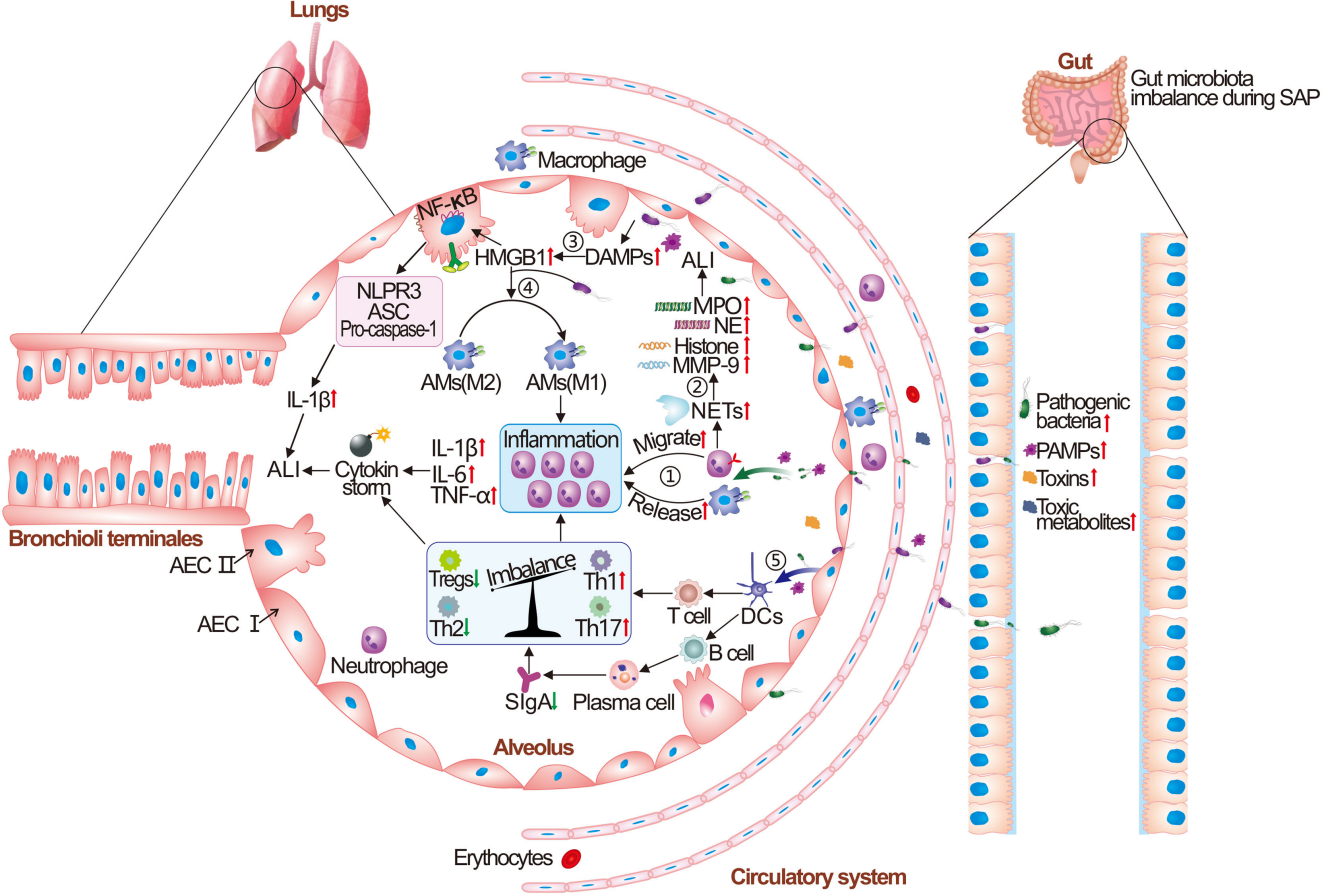
The pathogenesis of intestinal microbiota dysbiosis caused acute lung injury (ALI). Briefly, the intestinal pathogenic bacteria and harmful metabolites are translocated to the lung and recognized by pulmonary innate immune cells (AECs, AMs, DCs, and NK cells). Then, the release of persistent pro-inflammatory cytokines is induced by binding to their pattern recognition receptors (PRRs) on the cell surface, causing an “inflammatory storm”. A large number of neutrophil cells aggregate in the alveoli and neutrophil extracellular trap network (NETs) lead to increased release of neutrophil elastase (NE), myeloperoxidase (MPO), and histone. The inflammatory factors, granulins, and infiltrated red blood cells from vascular cause the damage and hemorrhage of lung tissue. Subsequently, the lung tissue initiates the immune responses of damage-associated molecular patterns (DAMPs), causing increased release of high mobility group protein B1 (HMGB1), the abnormal polarization of M1-type macrophages, and release of NOD-like receptor thermal protein domain associated protein 3 (NLRP3). Finally, lung tissue initiates an adaptive immune response, and increased production of pro-inflammatory Th1 and Th17 immune cells and suppression of Tregs cells could be observed, resulting in the paralysis of the immune system and irreversible ALI. AECs, alveolar epithelial cells; AMs, alveolar macrophages; DCs, dendritic cells; ASC, Apoptosis-associated speck-like protein containing a caspase recruit domain; SIgA, secretory IgA.

From a phylogenetic viewpoint, both the gut and lungs develop from the endoderm and connect to the outside environment, where the epithelial barrier and microbiota synergistically work to protect against pathogens (88). The crosstalk between the gut microbiota and the lung is referred to as the gut-lung axis, and its bidirectional regulation is critical for maintaining the host immune homeostasis as well as dynamic balance. Soluble microbial components and their metabolites, which are transported along with the circulatory system, are key mediators by which the intestinal microbiota and the lungs communicate with each other (89). Disturbed intestinal microbiota during SAP could cause PALI through a variety of mechanisms, as shown in **Figure 4**.

### Intestinal Bacterial Translocation-Mediated PALI

The disruption of gut barrier integrity during SAP leads to the translocation of pathogenic bacteria and their PAMPs to lung tissue, which can directly cause the release of inflammatory cytokines (TNF-α, IL-1β, and IL-6) from lung monocytes and macrophages, and subsequently act on the lung capillaries, alveolar epithelial cells (AECs), and the fluid layer on the alveolar surface to cause extensive ALI. Then, the DAMPs could be initiated in the lung tissue that causes a cascade reaction, including the extracellular migration of pro-inflammatory factors such as high mobility group box-1 (HMGB1), heat shock proteins (HSPs), and their bindings to the receptor for advanced glycation end products (RAGE), Toll-like receptor2 (TLR-2), and Toll-like receptor4 (TLR-4) on the surface of AECs. It will create a positive feedback loop that further leads to the upregulation of necrosis, apoptosis, and edema in the lungs (90–92). Elevated HMGB1 levels in lung tissue and bronchoalveolar lavage fluid (BALF) are demonstrated to be positively correlated with the level of pro-inflammatory cytokines using the PALI mice model. The PALI mice model was established by giving them 4g/kg intraperitoneal injection of L-arginine every hour for 2 hours, and the underlying mechanisms are linked to the activation of the TLR4/MyD88/NF-κB pathway and release of NLRP3 inflammasomes (93–96).

Neutrophil extracellular traps (NETs) are additional reported mediators correlated with the severity of decease and microbial diversity in ALI patients (97). NETs are decompressed chromatin and encapsulated granule proteins containing net-like structures that are released outside the cell from neutrophils stimulated by bacteria, PAMPs, etc. (98). NETs have the capacity to trap bacteria and other pathogens, but excessive activation could lead to the PALI and MODS by disrupting the endothelial/epithelial cell integrity, the intercellular junctions, and the functions of macrophages in the lungs. Pulmonary capillary microthrombosis could be promoted as well, mediated by the influence on intercellular communication, upregulation of matrix metalloproteinase-9 (MMP-9) expression, and release of inflammatory factors (15, 99–103). Furthermore, the administration of HMGB1 could stimulate the endoplasmic reticulum stress (ERS) and induce PALI by stimulating the formation of NETs (104).

### Alterations in the Functions of Alveolar Macrophages

**Figure 5:**
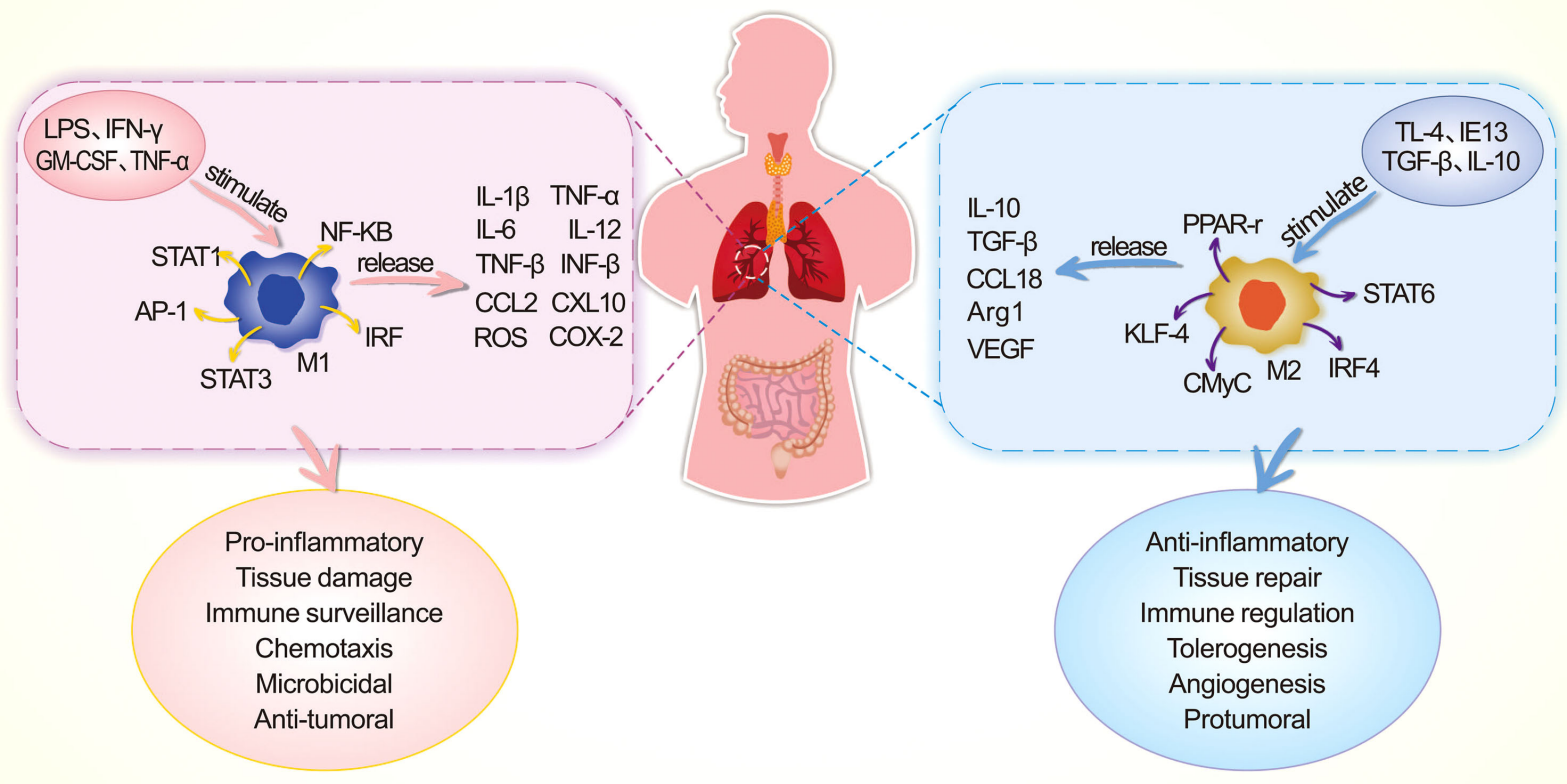
Comparison of M1-type macrophages and M2-type macrophages, in terms of stimulatory factors, transcription factors, released mediators, and functional properties during the process of PALI. AP-1, activating protein-1; ARG1, arginase 1; CMyC, transcription factor CMyC; GM-CSF, granulocyte-macrophage colony-stimulating factor; IFN-γ, interferon γ; IRF, interferon regulatory factor; KLF-4, krüppel-like factor 4; LPS, lipopolysaccharide; NF-κB, nuclear transcription factor κB; ROS, reactive oxygen species; STAT, signal transduction and activation of transcription factor; TGF-β, transforming growth factor ß; VEGF, vascular endothelial growth factor.

AMs, which are widely distributed in the pulmonary mesenchyme, perform the functions of phagocytosis, chemotaxis, immunity, and secretion. They play important roles in the defense against pathogens that cause PALI. Macrophages are presented in alveoli and part of macrophages in the lung interstitium are differentiated from monocytes; both of them constitute the innate immune functions in the lung. In a healthy state, macrophages in the alveoli maintain normal immunological function, while in the case of an immune imbalance (such as SAP/PALI), some macrophages tend to polarize toward M1-type macrophages which leads to fewer M2-type macrophages and excessive inflammatory responses (in **Figure 5**). M1-type AMs may be able to regulate the inflammatory responses and tissue damage by recruiting and activating the neutrophils and mononuclear phagocytes through secreting pro-inflammatory cytokines. M2-type AMs could release inhibitory cytokines that mediate the anti-inflammatory effects and tissue repair. Abnormal differentiation of AMs to the M1-type might induce severe inflammatory responses as the “catalyst” in the pathogenesis of PALI (105–107). Gram-negative bacteria are regarded as M1-type AM inducers, and the translocation of intestinal bacteria (such as *Escherichia coli* and *Shigella*) during SAP could result in the polarization of AMs to M1 type (108, 109). M1-type AMs polarization is linked to abnormal activation of the TLR4/NF-κB pathway, and M2-type AMs polarization is regulated by the IL-4/peroxisome proliferators-activated receptor-γ (PPAR-γ) pathway (105). Apart from this, HMGB1 could trigger PALI by inducing the polarization of AMs to M1 type, the activation of inflammatory vesicles in AMs, and the regulation of the RAGE/NF-κB signaling pathway (110).

Pyroptosis is programmed cell death mediated by cysteinyl aspartate-specific proteinase-1 (caspase-1), which is associated with the progression of PALI, as well as a reduction in SCFAs producing bacteria in the intestine (111). The “waterfall” inflammatory responses during PALI stimulate the swelling, rupture, and perforation of AMs. Then, a large amount of pro-inflammatory factors could be released, and the pyroptosis program is initiated by the abnormal activation of MAPK (112). Besides, the severity of PALI is linked to the exosomes generated by lung cells. Neutrophil-derived exosome miR-30d-5p could induce the polarization of AMs to M1-type, leading to the pyroptosis of AMs *via* the NF-κB signaling pathway (113). In addition, extracellular vesicles (EVs) released by macrophages can promote the polarization of M1-type AMs, causing the injury to the lungs (114). In contrast, the AECs-derived exosome miR-27b-3p can impact AMs functions by targeting RGS1-mediated calcium signaling-dependent immune responses (115).

### Activation of Abnormal Innate Immune Responses

Abnormal activation of the innate immune system in the lungs plays a decisive role in the pathogenesis of PALI (116). AECs, AMs, innate lymphoid cells (ILCs), natural killer cells (NKs), DCs, etc., of the pulmonary innate immune system can identify PAMPs and defend against the foreign pathogenic bacteria. Multiple targets are involved in the recognition of PAMPs (TLRs, NLRs, ALRs, etc.), which can result in the production of inflammatory factors, chemokines, interferons, reactive oxygen species (ROS), and others, thereby inducing the pro-inflammatory immunity (117). The increase in pathogen load or the release of “waterfall” inflammatory factors, in turn, stimulates the pulmonary adaptive immune system, in which T and B cells are activated and work together with the innate immune response in order to release SIgA, defensins, lysozyme, and complement (C3a, C5a), which then clear the pathogenic bacteria. Irreversible PALI is caused by the immune system paralysis for the imbalance between Th17 and Tregs, the increased antibody dependence-enhancement, the abnormal increase in complement, and the inactivation of innate immune cell function (116, 118, 119).

In addition, pathogenic bacteria-induced abnormal activation of DCs can increase the expression of intercellular cell adhesion molecule-1 (ICAM-1), and endothelial activation and abnormal immune responses are promoted by neutrophil infiltration, resulting in PALI (120). Facultative anaerobes can cause single amino acid nitration of Ras homolog gene family member A (RhoA) in PALI, resulting in the increased migration of immune cells along with the endothelial cell permeability (121). It is also reported that HMGB1 may exacerbate the bacterial endotoxin-induced PALI by regulating the cellular autophagy in DCs *via* Tregs inhibition and activating the PI3K/Akt/mammalian target of the rapamycin (mTOR) pathway (122, 123). Certain immune cells (ILC2s, ILC3s, Th17, etc.) migrating to the lungs (from the intestine) may play a direct role in the mediation of the abnormal immune responses during PALI (124).

### Metabolites From Intestinal Bacteria Mediate PALI

Several metabolites produced by the intestinal bacteria have been found to play a role in the mediation of the pathological process of PALI. Firstly, bacteria-derived LPS can induce PALI by activating NF-κB and MAPK pathways, and reported changes in metabolomics are also associated with abnormal fatty acid, phospholipid, and bile acid pathways (125). Free fatty acids (FFA) levels, a metabolite of triglycerides (TG), are found to be considerably higher in the serum of PALI mice, and FFAs are shown to induce the formation of NETs as well as activate DCs and T cells in the lungs (126, 127). Besides, the bacterial-derived metabolites p-cresol sulfate (PCS) and trimethylamine N-oxide (TMAO) are potential molecular markers for ERS in the lungs (128). Yumeng Huang et al. claimed that increased bacteria-derived 5-hydroxytryptophan (5-HT) in the peripheral circulation activates the formation of pulmonary NETs as well (129). While, several metabolites from intestinal bacteria, such as bile acids (ocaliva and ursodeoxycholic acid), butyric acid, etc., are proven to have protective effects on PALI (130–132).

In summary, the crosstalk between the intestinal microorganisms/bacterial metabolites and the inflammatory storm of the immune system in the lungs jointly lead to the development of PALI. The better understanding of the microbiota-gut-lung axis will aid in the elucidation of pathogenesis in PALI and provide theoretical foundations for the treatment of PALI by regulating intestinal microecology.

## Interventions by Regulating the Intestinal Microbiota

Due to the roles of intestinal microbiota in SAP, it has become a prospective therapeutic target for the treatment of PALI. In recent years, several strategies mediated by modulating the intestinal microecology or corresponding functions have been applied by clinicians, which not only reduce the in-hospital mortality of SAP patients but also minimize the risks of MODS to a certain extent (133–135). Herein, clinical and basic studies on the supplementation of probiotics/prebiotics, antibiotics treatment, fecal microbiota transplantation (FMT), the intervention of medication, and early enteral nutrition (EN) are discussed.

### Probiotics and Prebiotics

**Figure 6:**
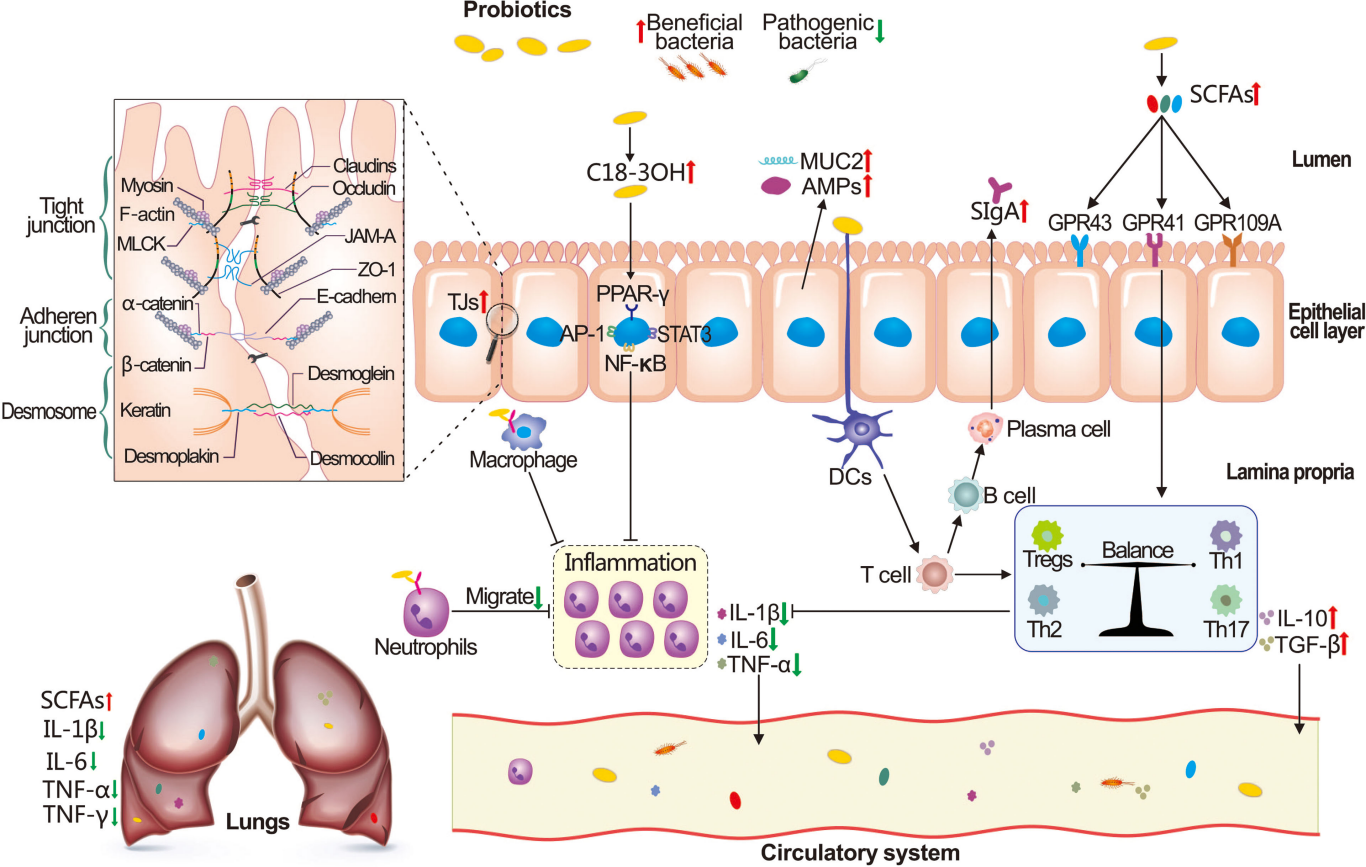
Possible mechanisms of probiotics supplementation on PALI recovery. Probiotics can restore the function of the intestinal barrier by inhibiting the proliferation of pathogenic bacteria, increasing the production of 3-hydroxyoctadecenoic acid (C18-3OH), short-chain fatty acids (SCFAs), antimicrobial peptides (AMPs), mucin2 (MUC2), and secretory IgA (SIgA), and regulating the expression of tight junction (TJ) proteins. In addition, probiotics have the capacity to reduce the neutrophil infiltration and release of inflammatory cytokines, maintain the balance of the intestinal immune system, and reduce the translocation of bacteria, inflammatory factors, etc., resulting in the alleviation of PALI.

Probiotics and prebiotics have been shown in recent years to have potential benefits in a variety of clinical conditions. However, the use of probiotics in the treatment of SAP remains debatable (136–140). It has been reported that probiotics and prebiotics can enhance intestinal barrier functions and strengthen the mucosal immune system. The underlying mechanisms are linked to the upregulation of TJ proteins, inhibition of pathogenic bacteria, and the induction of mucin2 (MUC2), AMPs, and SCFAs, as well as the reconstruction of the intestinal microenvironment (mediated by PRRs, etc.), as illustrated in **Figure 6** (141–145). Pujo et al. pointed out that probiotics produced 3-hydroxyoctadecenoic acid (C18-3OH), which could regulate the inflammatory response by binding to the PPAR-γ in intestinal epithelial cells, mediated by AP-1 (active protein-1), NF-κB, STAT3 (signal transducer and activator of transcription 3), and macrophage differentiation during PALI (146). Additionally, *Lactobacillus royale*, *Saccharomyces boulardii*, and *Bifidobacterium* have been reported to inhibit BT and decrease the incidence of PALI (46, 147–150). A double-blind clinical trial demonstrated that the time of hospitalization and abdominal pain is shortened in SAP patients following *Bacillus subtilis* and *Enterococcus faecalis* supplement (151). Similar to probiotics, prebiotics such as galactooligosaccharides, lactosucrose, and inulin-type fructan can substantially repair the functions of the intestinal barrier in SAP rats and have shown therapeutic potential in PALI (152–155).

Some studies, on the other hand, propose the opposite results. For instance, Gou et al. conducted a randomized controlled trial and concluded that probiotics have no beneficial impact on the 536 SAP patients (156). In addition, a double-blind trial showed that treatment with probiotics for 7 days did not present an advantage on the intestinal permeability as well as endotoxemia in SAP patients (157). The authors conclude that the heterogeneity of probiotics observed during SAP attributes to multiple factors such as the pancreatitis etiology, type of probiotics, intervention time of treatment, and the individuation in gut microbiota (158–160).

### Antibiotics

Antibiotics are commonly used by clinicians for prophylactically inhibiting the growth of pathogenic bacteria in SAP patients and reducing secondary SAP infection complications. Previous studies have found that clearance of intestinal bacteria with a combination of broad-spectrum antibiotics can reduce the severity of PALI in mice. However, the lethal effect on specific intestinal bacteria might result in the risk of fungal infections along with the rapid proliferation of drug-resistant bacteria, fueling heated debates regarding antibiotic usage in pancreatic surgery (22, 161).

A study by Soares et al. revealed that the use of meropenem accelerates the progress of SAP and it causes the multi-drug resistant BT (162). Jacobs et al. observed the sustaining high levels of IL-6 in alveolar lavage fluid of PALI mice after 2 weeks of therapy with advanced antibiotics (163). The latest World Society of Emergency Surgery (WSES) guidelines for the management of SAP report that the prophylactic use of antibiotics in patients with AP is not associated with a reduction in mortality or morbidity (164). Hence, antibiotics are no longer recommended as a priority for SAP patients with the exception of obvious infections. And the choice of broad-spectrum antibiotics should take the bacteriological changes in SAP and their pharmacokinetics into account, and further studies must be conducted (165, 166).

### FMT

In clinical practice, FMT has been developed as a second-line treatment option for recurrent infection of *Clostridium Difficile*, and it has now become a treatment option for a variety of diseases including IBD, irritable bowel syndrome (IBS), autism, hepatic encephalopathy, etc. (167–170). Even though evident changes have been observed in SAP, the identification of beneficial strains, antibiotic-resistant strains, and detrimental strains remains a challenge, which creates a gap in the protection against PALI by FMT. Furthermore, the lack of standard in FMT is another question that limits its use. A randomized controlled clinical trial by Ding et al. pointed out that FMT could not reduce the complications in SAP patients and that there is a risk of disruption in the intestinal barrier (171). Yin et al., on the other hand, found that FMT attenuates the LPS-induced PALI in rats by inhibiting the PI3K/AKT/NF-κB signaling pathway, the mechanisms of which are associated with the expression of ICAM-1 (172, 173). Since the current findings are primarily limited to animal studies, more evidence is needed to further assess the benefits of FMT in clinical usage.

### Traditional Chinese Medicine

TCM has its unique advantages in the treatment of SAP, and herbal formulas such as Da Cheng Qi Decoction, Chaiqin Chengqi Decoction, Da Chaihu Decoction, etc., have been extensively utilized in SAP patients (174). Our previous studies showed that emodin, the main active component in Rheum officinale, could alleviate PALI by targeting the cold-inducible RNA-binding protein (CIRP)/NLRP3/IL-1β/CXCL1 pathway and the inhibition of neutrophil protease (5, 175). Because the majority of the herbal drugs are taken orally, the components inevitably pass through the digestive tract and reach the intestine, allowing for interactions with the intestinal microbiota. Piao et al. argued that picroside II increases the abundance of *Lactobacillus*, *Prevotella*, and restores the intestinal barrier by inhibiting the TLR4-dependent PI3K/AKT/NF-κB pathway, which thereby alleviates SAP (11) (176). Moreover, emodin has been reported to repair intestinal barrier functions and alleviate PALI by upregulating SCFA-producing bacteria (such as *Akkermansia Muciniphila* and *Bacteroides*) (177).

### Enteral Nutrition

In clinical treatment, patients with SAP are frequently advised to fast and decompress the gastrointestinal tract to lower the burden on the pancreas. Long-term enteral nutrition deprivation in SAP patients, on the one hand, will lead to the reduction of digestive fluid (bile, intestinal fluid, lysozyme, etc.) and accelerate the injury of the intestinal barrier; on the other hand, it will cause intestinal mucosal epithelial necrosis and atrophy of intestinal villi, which further increases the risk of PALI (178). However, in order to maintain the function of the intestine, large amounts of fluids (including crystalloids, colloids, electrolytes, etc.) neglect the possible secondary ischemia/reperfusion (I/R) injury. And I/R injury in SAP leads to the increase of intestinal permeability as well as the disruption of intestinal microecological balance, all of which can contribute to BT risks and inflammatory responses (179). As a result, the current guidelines for the management of SAP recommend that patients should be given enteral nutrition (by intranasal tube or oral administration) at the early stage rather than total parenteral nutrition (TPN), so as to preserve the intestinal functions and prevent the secondary infectious complications (164). Early nutrition, according to Zhao et al., shortens the length of stay in hospital along with the occurrence of PALI in patients (180). Sun et al. found that early enteral nutrition affects the functions of the intestinal immune system, thereby reducing pancreatic necrosis and systemic inflammatory responses (181). Furthermore, glutamine supplementation is reported to be related to improved intestinal permeability and oxidative stress in SAP patients (182).

## Summary and Prospections

Given the critical role of the microbiota-gut-lung axis in the pathogenesis of PALI, gut microbiota, regarded as a “bridge” in the pathogenesis of PALI, has been increasingly emphasized. In this paper, we summarized the dysbiosis process in the abundance of intestinal microbiota under SAP as well as the possible mechanisms by which intestinal microecological disorders accelerate bacterial translocation through disruption of the intestinal physical, chemical, and immune barriers to cause PALI. In particular, the PAMPs-mediated immunol mechanisms in the occurrence of PALI by binding with pattern recognition receptors (PRRs) *via* the microbiota-gut-lung axis are taken into account. Finally, we analyze several potential effective interventions that may alleviate PALI by regulating the intestinal microbiota, and it is expected to provide further references for clinical applications. Many questions still need to be addressed and further studies on the molecular mechanisms of the bidirectional communications between the intestinal microbiota and the lungs remain to be investigated. Moreover, inhibition of bacterial translocation by restoring the homeostasis of the intestinal microbiota may be a promising therapeutic target for protecting against PALI. The exploration of bacterial markers for early diagnosis of PALI is another promising subsequent topic in this field. More cohort studies and optimally designed clinical trials regarding intestinal microbiota interventions are necessary for the purpose of evaluating the safety and therapeutic efficacy of PALI treatment. Furthermore, the findings on the microbiota-gut-lung axis are expected to be useful for the diagnosis and prognosis of PALI.

## Author Contributions

ZW and FL wrote the manuscript; ZW, YL, and SM made the figures; JL, HG, QY, SM, and CX revised the manuscript; HC designed the manuscript. All authors have read and approved the final manuscript.

## Funding

This study was supported by the National Key R & D Programmes of China (No. 2019YFE0119300), the National Natural Science Foundation of China (No. 82074158 and No.82104594).

## Conflict of Interest

The authors declare that the research was conducted in the absence of any commercial or financial relationships that could be construed as a potential conflict of interest.

## Publisher’s Note

All claims expressed in this article are solely those of the authors and do not necessarily represent those of their affiliated organizations, or those of the publisher, the editors and the reviewers. Any product that may be evaluated in this article, or claim that may be made by its manufacturer, is not guaranteed or endorsed by the publisher.

## Glossary

**Table:**

AECs	alveolar epithelial cells
AhR	arylhydrocarbon receptor
ALI	acute lung injury
ALRs	melanoma 2 (AIM)-like receptors
AMPs	antimicrobial peptides
AMs	alveolar macrophages
AP-1	active protein-1
BALF	bronchoalveolar lavage fluid
BT	bacterial translocation
C18-3OH	3-hydroxyoctadecenoic acid
caspase-1	cysteinyl aspartate-specific proteinase-1
CIRP	cold-inducible RNA-binding protein
DAMPs	damage-associated molecular patterns
DCA	deoxycholic acid
DCs	dendritic cells
EN	enteral nutrition
ERS	endoplasmic reticulum stress
EVs	extracellular vesicles
FFAs	free fatty acids
FMT	fecal microbiota transplantation
FXR	farnesoid X receptor
GALTs	gut-associated lymphoid tissues
GCs	goblet cells
GF	germ-free
5-HT	5-hydroxytryptophan
HMGB1	high mobility group box-1
HSPs	heat shock proteins
I/R	ischemia/reperfusion
IBS	irritable bowel syndrome
ICAM-1	intercellular cell adhesion molecule-1
IECs	intestinal epithelial cells
IELs	intestinal intraepithelial lymphocytes
IFN-γ	interferon γ
ILCs	innate lymphoid cells
LPLs	lamina propria lymphocytes
MAPK	mitogen-activated protein kinase
MLNs	mesenteric lymph nodes
MMP-9	matrix metalloproteinase-9
MODS	multiple organ dysfunction syndromes
MOF	multiple organ failure
mTOR	mammalian target of rapamycin
NETs	neutrophil extracellular traps
NF-&κB	nuclear factor-κB
NKs	natural killer cells
NLRs	nucleotide-binding oligomerization domain like receptors
NLRP3	NOD-like receptor thermal protein domain associated protein 3
PAFr	platelet-activating receptor
PALI	SAP-associated lung injury
PAMPs	pathogen-associated molecular patterns
PCs	paneth cells
PCS	p-cresol sulfate
PP	peyer patch
PPAR-γ	peroxisome proliferators-activated receptor-γ
PRRs	pattern recognition receptors
RAGE	receptor for advanced glycation end products
RhoA	Ras homolog gene family member A
SAP	severe acute pancreatitis
SCFAs	short-chain fatty acids
SDD	selective digestive decontamination
SIgA	secretory IgA
SIRS	systemic inflammatory response syndrome
STAT3	signal transducer and activator of transcription 3
TCM	traditional Chinese medicine
TG	triglycerides
TJs	tight junctions
TLRs	toll-like receptors
TMAO	trimethylamine N-oxide
TPN	total parenteral nutrition
Tregs	regulatory T cells
vWF	vascular hemophilia factor
WSES	World Society of Emergency Surgery
ZO-1	zonula occludens-1
